# Chronic Cannabidiol Administration Fails to Diminish Blood Pressure in Rats with Primary and Secondary Hypertension Despite Its Effects on Cardiac and Plasma Endocannabinoid System, Oxidative Stress and Lipid Metabolism

**DOI:** 10.3390/ijms21041295

**Published:** 2020-02-14

**Authors:** Patryk Remiszewski, Iwona Jarocka-Karpowicz, Michał Biernacki, Anna Jastrząb, Eberhard Schlicker, Marek Toczek, Ewa Harasim-Symbor, Anna Pędzińska-Betiuk, Barbara Malinowska

**Affiliations:** 1 Department of Experimental Physiology and Pathophysiology, Medical University of Białystok, 15-222 Białystok, Poland; patryk.remiszewski@umb.edu.pl (P.R.); marek.toczek@umb.edu.pl (M.T.); aniutka@umb.edu.pl (A.P.-B.); 2 Department of Analytical Chemistry, Medical University of Białystok, 15-222 Białystok, Poland; iwona.jarocka-karpowicz@umb.edu.pl (I.J.-K.); michal.biernacki@umb.edu.pl (M.B.); anna.jastrzab@umb.edu.pl (A.J.); 3 Department of Pharmacology and Toxicology, University of Bonn, 53127 Bonn, Germany; e.schlicker@uni-bonn.de; 4 Department of Physiology, Medical University of Białystok, 15-222 Białystok, Poland; eharasim@umb.edu.pl

**Keywords:** 2-arachidonoylglycerol, anandamide, cannabidiol, cannabinoid receptor, SHR, DOCA-salt, endocannabinoids, oxidative stress

## Abstract

We investigated the influence of cannabidiol (CBD) on blood pressure (BP) and heart rate (HR) in spontaneously (SHR) and deoxycorticosterone (DOCA-salt) hypertensive rats. Hypertension was connected with increases in cardiac and plasma markers of lipid peroxidation in both models, whereas cardiac endocannabinoid levels decreased in SHR and increased in DOCA-salt. CBD (10 mg/kg once a day for 2 weeks) did not modify BP and HR in hypertension but counteracted pro-oxidant effects. Moreover, it decreased cardiac or plasma levels of anandamide, 2-arachidonoylglycerol and oleoyl ethanolamide in DOCA-salt and inhibited the activity of fatty acid amide hydrolase (FAAH) in both models. In the respective normotensive control rats, CBD increased lipid peroxidation, free fatty acid levels and FAAH activity. In conclusion, chronic CBD administration does not possess antihypertensive activity in a model of primary and secondary (DOCA-salt) hypertension, despite its antioxidant effect. The latter may be direct rather than based on the endocannabinoid system. The unexpected CBD-related increase in lipid peroxidation in normotensive controls may lead to untoward effects; thus, caution should be kept if CBD is used therapeutically.

## 1. Introduction

Cannabidiol (CBD) is one of the most abundant cannabinoids derived from the *Cannabis sativa* plant and devoid of a psychoactive effect [1,2]. CBD binds to cannabinoid CB_1_ and CB_2_ receptors with much lower affinity than Δ^9^-tetrahydrocannabinol (THC) [3] and interacts with GPR18, GPR55 and TRPV1 receptors [4]; it possesses a very marked antioxidant effect [5,6,7]. CBD is licensed for the treatment of some types of childhood epilepsy (Dravet and Lennox-Gastaut syndrome) in the United States [4,8] and, in combination with THC, for the treatment of multiple sclerosis-associated spasticity in Canada and in the European Union [4]. In addition, a potential therapeutic action of CBD is being considered in anxiety disorders, schizophrenia, depression, Alzheimer’s disease, Parkinson’s disease, pain, cancer, inflammatory and autoimmune diseases and diabetic complications [2,4,9].

CBD may become a strategy also for the treatment of cardiovascular diseases, including hypertension [3,9]. To date, blood pressure-lowering effects of CBD were observed under stress conditions in humans [10,11,12] and in stressed animals [13,14]. However, the effect of CBD on the blood pressure of hypertensive individuals has been studied in one study only; in a paper on conscious spontaneously hypertensive rats [15], a single intraperitoneal dose of CBD (10 mg/kg) failed to affect blood pressure.

Hypertension is a disease with a complex pathomechanism, which includes, among others, changes in the endothelium and redox balance, both within the heart and blood vessels [16,17]. CBD is suggested to be a potential positive modulator of hypertension thanks to its vasodilatory action [3,9,11,18]. Another property that may be of key importance in a potential antihypertensive activity of CBD is its impact on oxidative stress. Attenuation of oxidation and/or nitration parameters by CBD was observed in acute experiments on human endothelium cells treated with high glucose [19], on the liver of mice subjected to ischemia/reperfusion [20] and on mouse hippocampal cells subjected to oxygen plus glucose deprivation/reperfusion [21]. Similar beneficial effects were also obtained in chronic experiments on the heart [22] and retina [23] from diabetic mice, on mouse hepatic cells with ethanol-induced liver injury [24,25], on the heart from doxorubicin-treated mice [26] and rats [27] and on the heart and other tissues of rats with sepsis [28].

The mechanism of CBD in the latter studies is complex and probably results from direct antioxidant properties [3,29] but may also be related to an effect on the endocannabinoid system, which is important for the modulation of oxidative stress [30,31]. CB_1_ receptors are mainly associated with its promotion [32,33,34], whereas CB_2_ [35,36,37,38,39] and GPR18 [40,41] receptors reduce oxidation parameters in cardiovascular system including heart. There are contradictory reports regarding modulation of oxidative stress by TRPV1 and GPR55 receptors [30,31].

Although CBD probably does not work via endocannabinoid receptors directly, it may act through augmentation of endocannabinoid tone [42]. CBD inhibits fatty acid amide hydrolase (FAAH) [43] and can interact with the anandamide membrane transporter [44,45] both of which may increase levels of endocannabinoids and related lipids. They may have positive effects and be used as a target in pharmacotherapy [46] but in some cases, can also exert untoward actions [47]. In this context, one should keep in mind that the FAAH inhibitor URB597 and hypertension may affect cardiac and plasma oxidative stress, endocannabinoid levels and lipid metabolism in a model-dependent manner [48,49].

The first aim of this study was to investigate whether chronic, unlike acute [15], administration of CBD reduces blood pressure (BP) and heart rate (HR) in rats with primary and secondary hypertension. Moreover, we studied whether CBD has an impact (ii) on the redox system, (iii) the endocannabinoid system in heart and plasma and (iv) free polyunsaturated fatty acids (PUFAs) and phospholipid PUFAs.

## 2. Results

### 2.1. General

**Figure 1:**
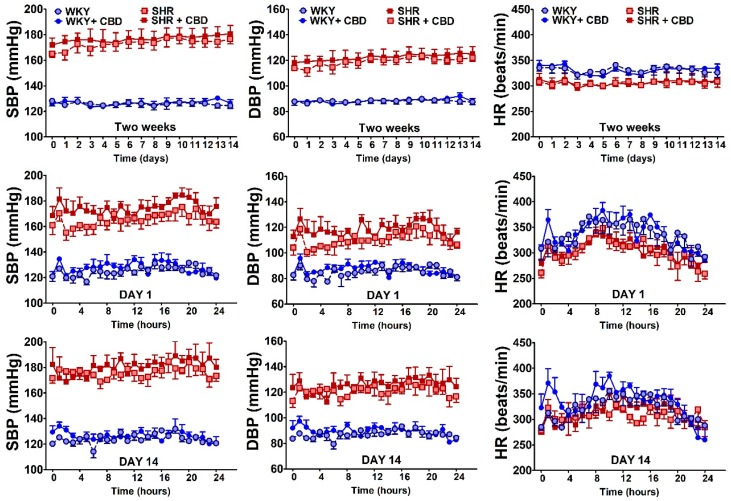
Influence of cannabidiol (CBD) or its vehicle on systolic blood pressure (SBP), diastolic blood pressure (DBP) and heart rate (HR) in spontaneously hypertensive rats (SHR) and their normotensive controls (WKY). Parameters were measured telemetrically every hour (shown for DAY 1 and 14 in the intermediate and bottom panels, respectively) and means for each day were determined (shown in the top panels). CBD (10 mg/kg) was injected i.p. once daily for 14 days (control animals received the vehicle for CBD instead); first and final injection on DAY 1 and 14, respectively. Data are expressed as the median with interquartile range; *n* = 4. All values of SBP and DBP in SHR (in the presence of CBD or its vehicle) were higher than their respective values in WKY (*p* < 0.001), and values of HR in SHR were lower than in WKY (*p* < 0.001 with 2 exceptions at the 14th day: WKY vs. SHR *p* < 0.05 and WKY + CBD vs. SHR + CBD: *p* < 0.01).

As shown in Table 1 and Figure 1 (in which cardiovascular parameters were measured by the non-invasive method and telemetrically, respectively) SBP and DBP, registered before the first administration of CBD or its vehicle, were higher in SHR and DOCA rats than in the respective control animals (WKY and SHAM). HR tended to be lower in DOCA compared to SHAM rats and higher in SHR than in WKY when non-invasive registration was used (Table 1); in animals with telemetrical registration, HR was higher in WKY than in SHR (Figure 1). Two-week administration of CBD 10 mg/kg did not affect SBP, DBP and HR in normo- or hypertensive rats. The vehicle for CBD decreased (or tended to decrease) HR by about 7%–8% in WKY and SHAM. Similarly CBD reduced HR by about 10% in WKY (Table 1). We did not observe any cardiovascular effect of CBD during 24-h observation on the first and the last day of its administration (Figure 1).

The body weight of SHR and DOCA rats was lower than that of WKY and SHAM rats, respectively, both before the first and after the final administration of the vehicle of CBD (Table 1). When animals were considered which had received CBD instead, differences were smaller or did not occur at all (Table 1).

### 2.2. Influence of Hypertension and CBD on the Endocannabinoid System

**Figure 2:**
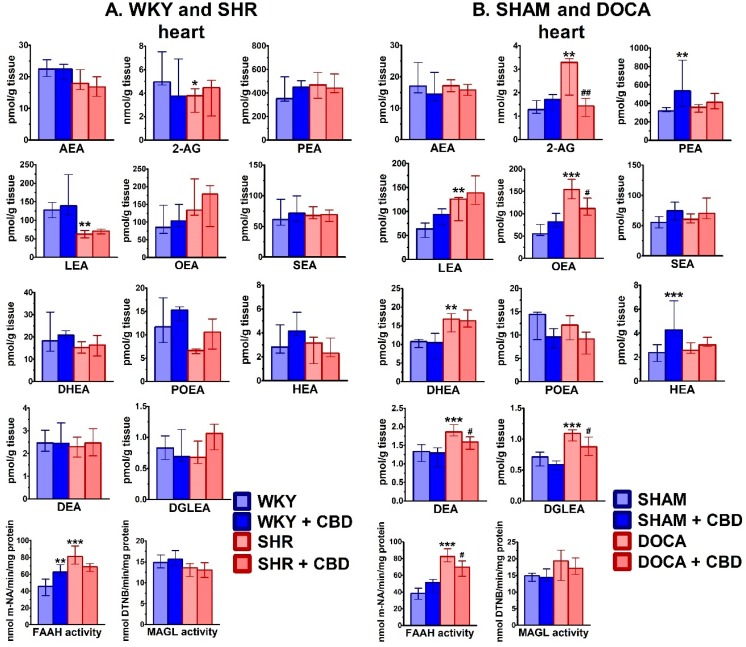
Influence of hypertension and cannabidiol (CBD) or its vehicle on endocannabinoid levels in heart isolated from spontaneously (SHR; **A**) and deoxycorticosterone-salt (DOCA; **B**) hypertensive rats and their normotensive controls (WKY (**A**) and SHAM (**B**), respectively). CBD (10 mg/kg) was injected i.p. once daily for 14 days; the respective controls received the vehicle of CBD instead; hearts were prepared 24 h after the final injection. Data are expressed as the median with interquartile range; *n* = 5-7; */^#^ *p* < 0.05; **/^##^ *p* < 0.01; *** *p* < 0.001 significantly different from WKY or SHAM (*) and from SHR or DOCA (#). AEA—anandamide; 2-AG—2-arachidonoylglycerol; PEA—palmitoyl ethanolamide; LEA - linolenoyl ethanolamide; OEA—oleoyl ethanolamide; SEA—stearoyl ethanolamide; DHEA—docosahexaenoyl ethanolamide; POEA—palmitoleoyl ethanolamide; HEA—homo-γ-linolenyl ethanolamide; DEA—docosatetraenoyl ethanolamide; DGLEA—dihomo-γ-linolenoyl ethanolamide; FAAH—fatty acid amide hydrolase; MAGL—monoacylglycerol lipase; m-NA—m-nitroaniline; DTNB—5,5′-dithiobis-2-dinitrobenzoic acid.

As shown in Figure 2, 2-AG showed the highest level among the endocannabinoids; values in the heart of normotensive WKY and SHAM rats (expressed as median (interquartile range)) amounted to 5.0 (4.7;7.5) (*n* = 7) and 1.3 (1.1;1.7) nmol/g tissue (*n* = 7), respectively. The levels of the best-known endocannabinoid AEA were relatively low, i.e., about 220 and 75 times lower than the respective values of 2-AG in WKY and SHAM rats. Although levels of PEA, LEA and OEA were higher than those of AEA, they were still lower than 2-AG levels by factors of about 15, 40 and 60 (WKY) and 5, 20 and 25 (SHAM), respectively. HEA, DEA and DGLEA showed the lowest levels, i.e., about 1–3 pmol/g tissue in all cases.

Hypertension modified cardiac endocannabinoid levels in a model-dependent manner (Figure 2). Thus, they mostly decreased (2-AG by 24% and LEA by 51%) or tended to decrease (AEA, DHEA POEA, HEA, DEA and DGLEA) in SHR in comparison to WKY. In contrast, they increased (2-AG by 158%, LEA by 97%, OEA by 180%, DHEA by 56%, DEA by 40% and DGLEA by 54%) in DOCA hearts in comparison to SHAM. Chronic CBD administration did not modify the cardiac endocannabinoid levels in SHR and WKY. However, it decreased levels of 2-AG (−57%), OEA (−28%), DEA (−15%) and DGLEA (−20%) in hearts of DOCA but increased PEA (+68%) and HEA (+80%) levels in SHAM rats.

In both models of hypertension, we observed increases in FAAH activities in comparison to the respective normotensive control (+77% in SHR and +115% in DOCA), but no changes in MAGL activities (Figure 2). CBD did not modify MAGL activity but decreased FAAH activity in DOCA and tended to do so in SHR. Moreover, it increased FAAH activity in WKY.

**Figure 3:**
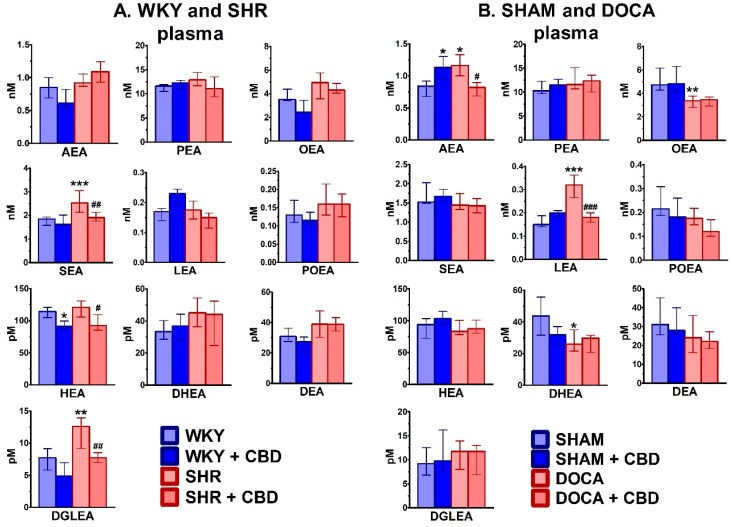
Influence of hypertension and cannabidiol (CBD) or its vehicle on endocannabinoid levels in plasma isolated from spontaneously (SHR; **A**) and deoxycorticosterone-salt (DOCA; **B**) hypertensive rats and their normotensive controls (WKY (**A**) and SHAM (**B**), respectively). CBD (10 mg/kg) was injected i.p. once daily for 14 days; the respective controls received the vehicle of CBD instead. Plasma was obtained 24 h after the final injection. Data are expressed as the median with interquartile range; *n* = 5–7; */^#^ *p* < 0.05; **/^##^ *p* < 0.01; ***/^###^ *p* < 0.001 significantly different from WKY or SHAM (*) and from SHR or DOCA (#). AEA—anandamide; PEA—palmitoyl ethanolamide; OEA—oleoyl ethanolamide; SEA—stearoyl ethanolamide; LEA—linolenoyl ethanolamide; POEA—palmitoleoyl ethanolamide; HEA—homo-γ-linolenyl ethanolamide; DHEA—docosahexaenoyl ethanolamide; DEA—docosatetraenoyl ethanolamide; DGLEA—dihomo-γ-linolenoyl ethanolamide.

Plasma AEA levels were about 1 nM both in WKY and SHAM rats (Figure 3). For both control groups, levels were higher (PEA and OEA by about 10 and 5 times, respectively) and similar (SEA) when compared to the values of AEA. In contrast to SHR hearts, the plasma endocannabinoid levels increased (SEA +38%, DGLEA +63%) or tended to increase (OEA, POEA DHEA and DEA) in comparison to WKY. In DOCA, plasma levels increased (AEA +38% and LEA +113%), decreased (OEA −29% and DHEA −41%) or tended to decrease (POEA and DEA) in comparison to SHAM rats.

Chronic CBD administration decreased plasma levels of SEA (−25%), HEA (−23%) and DGLEA (−39%) in SHR and AEA (−29%) and LEA (−44%) in DOCA or tended to do so in the case of PEA, OEA and LEA in SHR (Figure 3). Moreover, CBD decreased plasma levels of HEA (−20%) in WKY and increased the plasma level of AEA (+33%) in SHAM or tended to decrease levels of AEA, OEA, POEA, DEA and DGLEA in WKY and levels of POEA and DHEA in SHAM.

**Figure 4:**
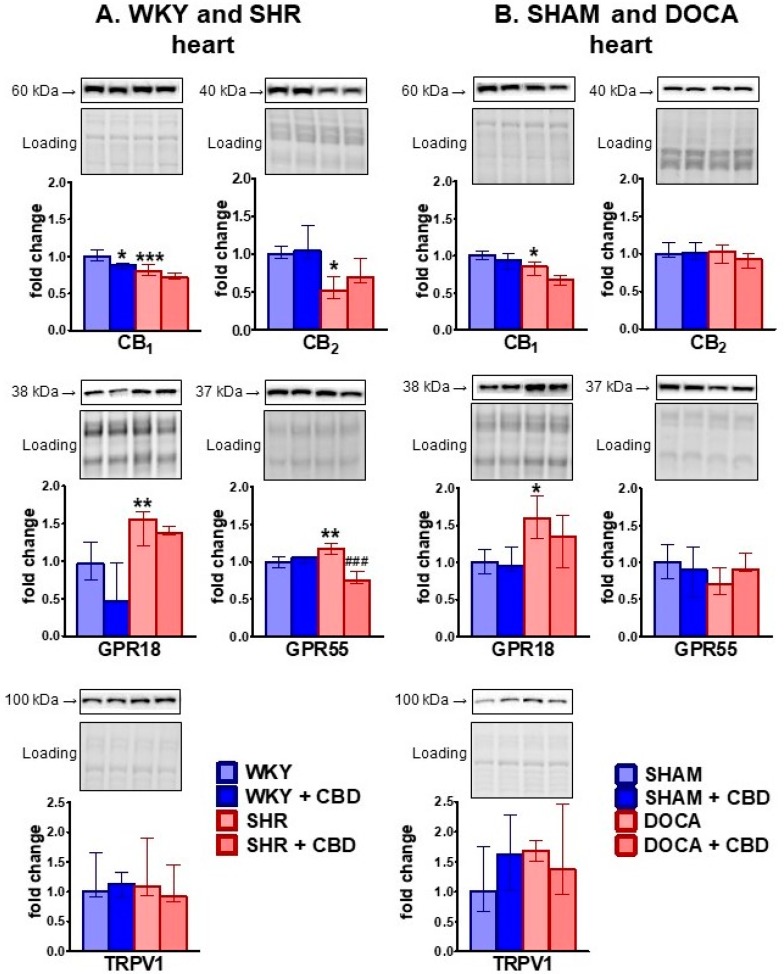
Influence of hypertension and cannabidiol (CBD) or its vehicle on expression of receptors in left ventricle isolated from spontaneously (SHR; **A**) and deoxycorticosterone-salt (DOCA; **B**) hypertensive rats and their normotensive controls (WKY (**A**) and SHAM (**B**), respectively). CBD (10 mg/kg) was injected i.p. once daily for 14 days; the respective controls received the vehicle of CBD instead. Hearts were prepared 24 h after the final injection. Receptor protein was determined by Western blots and given as fraction of the value in the normotensive control (first of the four columns). Images obtained using stain-free gel technology that allows for total protein visualization and quantification are shown as a loading control (Loading). Data are expressed as the median with interquartile range; *n* = 5–6; * *p* < 0.05; ** *p* < 0.01; ***/^###^ *p* < 0.001 significantly different from WKY or SHAM (*) and from SHR or DOCA (#).

In the cardiac left ventricle, cannabinoid CB_1_ and CB_2_ receptor density decreased but GPR18 and GPR55 density increased in SHR compared to WKY. In DOCA, compared to SHAM, only a decrease in CB_1_ density and an increase in GPR18 density occurred. The increase in GPR18 receptor density was particularly marked in both hypertension models (by about 60%) (Figure 4). Chronic CBD administration decreased GPR55 density in SHR and tended to decrease CB_1_ and GPR18 receptor density in SHR and CB_1_, CB_2_ and GPR18 receptor density in DOCA. Moreover, CBD decreased CB_1_ and tended to reduce GPR18 receptor density in WKY. TRPV1 receptor density was not modified by hypertension or CBD treatment (Figure 4).

### 2.3. Influence of Hypertension and CBD on Oxidative Stress

**Figure 5:**
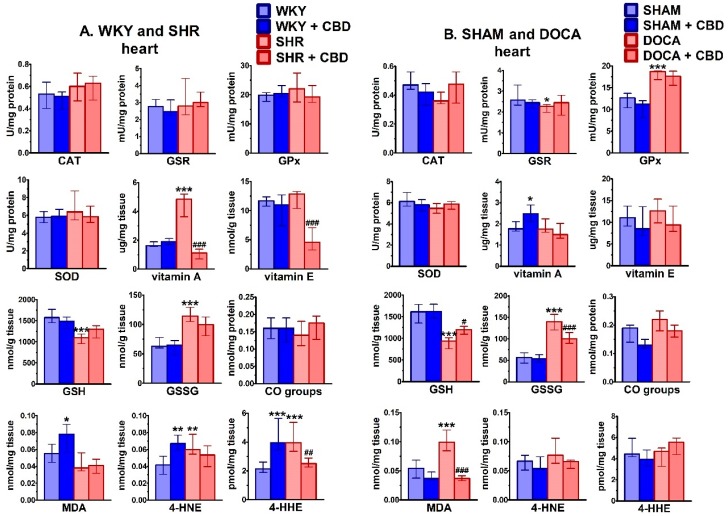
Influence of hypertension and cannabidiol (CBD) or its vehicle on activity/level of oxidative stress parameters in heart isolated from spontaneously (SHR; **A**) and deoxycorticosterone-salt (DOCA; **B**) hypertensive rats and their normotensive controls (WKY (**A**) and SHAM (**B**), respectively). CBD (10 mg/kg) was injected i.p. once daily for 14 days; the respective controls received the vehicle of CBD instead. Hearts were prepared 24 h after the final injection. Data are expressed as the median with interquartile range; *n* = 5–7; */^#^ *p* < 0.05; **/^##^ *p* < 0.01; ***/^###^*p* < 0.001 significantly different from WKY or SHAM (*) and from SHR or DOCA (#). CAT—catalase; GSR—glutathione-disulfide reductase; GPx—glutathione peroxidase; SOD—superoxide dismutase; GSH—glutathione; GSSG—glutathione disulfide; CO groups—protein carbonyl groups; MDA—malondialdehyde; 4-HNE—4-hydroxynonenal; 4-HHE—4-hydroxyhexenal.

As shown in Figure 5, activities of cardiac antioxidant principles underwent slight changes in hypertension. Thus, in comparison to the respective normotensive tissues, GPx activity increased or tended to increase in DOCA (+48%) and SHR, respectively, whereas GSR activity decreased (−12%) in DOCA heart. However, SOD and CAT were not altered in SHR and DOCA. The cardiac vitamin A (but not E) level increased in SHR (+198%), whereas the levels of both antioxidant vitamins did not change in the heart of DOCA rats. Levels of glutathione and glutathione disulfide were altered by both models of hypertension in the same way: GSH decreased in the heart of SHR (−30%) and DOCA (−42%) and GSSG increased (+82% and +149%, respectively). Products of lipid peroxidation increased in the heart of SHR (4-HNE +45% and 4-HHE +86%) and DOCA rats (MDA +82%). Changes in cardiac CO groups were neither observed in SHR nor in DOCA.

Chronic CBD administration was associated mainly with a decrease in cardiac levels of the products of lipid peroxidation (Figure 5). The effect was significant for 4-HHE (−37%) in SHR and for MDA (−63%) in DOCA and a tendency was observed in the case of 4-HNE in SHR and DOCA. Moreover, cardiac levels of vitamin A (−77%) and E (−64%) were reduced in SHR or tended to be so in DOCA. In addition, the level of GSH was increased (+29%) and that of GSSG was decreased (−29%) in DOCA; in SHR, both parameters tended to be altered Other cardiac parameters of oxidative stress were not modified by CBD in hypertensive rats (Figure 5). In normotensive rats, CBD increased levels of vitamin A in SHAM (+41%) and unexpectedly levels of products of lipid peroxidation (MDA (+42%), 4-HNE (+61%) and 4-HHE (+86%)) in WKY but not in SHAM (Figure 5).

**Figure 6:**
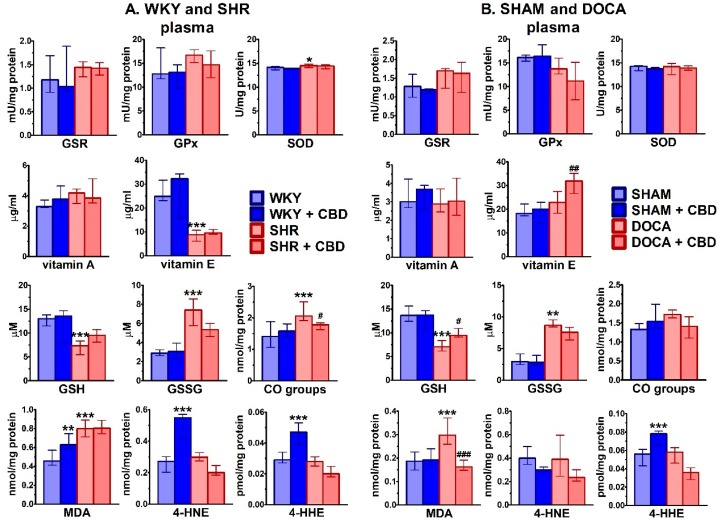
Influence of hypertension and cannabidiol (CBD) or its vehicle on activity/level of oxidative stress parameters in plasma isolated from spontaneously (SHR; **A**) and deoxycorticosterone-salt (DOCA; **B**) hypertensive rats and their normotensive controls (WKY (**A**) and SHAM (**B**), respectively). CBD (10 mg/kg) was injected i.p. once daily for 14 days; the respective controls received the vehicle of CBD instead. Plasma was obtained 24 h after the final injection. Data are expressed as the median with interquartile range; *n* = 5–7; */^#^ *p* < 0.05; **/^##^ *p* < 0.01; ***/^###^ *p* < 0.001 significantly different from WKY or SHAM (*) and from SHR or DOCA (#). GSR—glutathione-disulfide reductase; GPx—glutathione peroxidase; SOD—superoxide dismutase; GSH—glutathione; GSSG—glutathione disulfide; CO groups—protein carbonyl groups; MDA—malondialdehyde; 4-HNE—4-hydroxynonenal; 4-HHE—4-hydroxyhexenal.

Similarly to the heart, plasma activities of antioxidant principles underwent only small changes in hypertension in comparison to normotension (Figure 6). Thus, only in SHR SOD (+3%) and GPx activities increased or tended to increase, respectively. The level of vitamin E decreased in SHR (−65%), but was not modified in DOCA. Levels of vitamin A did not undergo changes in either hypertension model. Levels of GSH and GSSG were modified in a comparable way in SHR and DOCA, i.e., they decreased and increased by about 45% and 175%, respectively. Levels of MDA (but not of other products of lipid peroxidation) increased by about 65% both in SHR and DOCA. The CO group level increased in SHR (+46%) and tended to do so in DOCA.

Chronic CBD administration increased plasma levels of vitamin E (+39%) in DOCA. GSH was increased (+34%) in DOCA and tended to be increased in SHR whereas GSSG tended to decrease in either hypertensive rat model. For antioxidant enzyme activities only tendencies towards a decrease of GPx (SHR and DOCA) and GSR (DOCA) were observed. However, CBD reduced plasma levels of CO groups (-14%) in SHR and of MDA (-45%) in DOCA. Moreover, it tended to decrease plasma levels of 4-HNE (-32%) in SHR and 4-HHE (-37%) in DOCA. Unexpectedly it also increased plasma levels of MDA (+38%), 4-HNE (+101%) and 4-HHE (+62%) in WKY and of 4-HHE (+39%) in SHAM (Figure 6).

### 2.4. Influence of Hypertension and CBD on Lipids

**Figure 7:**
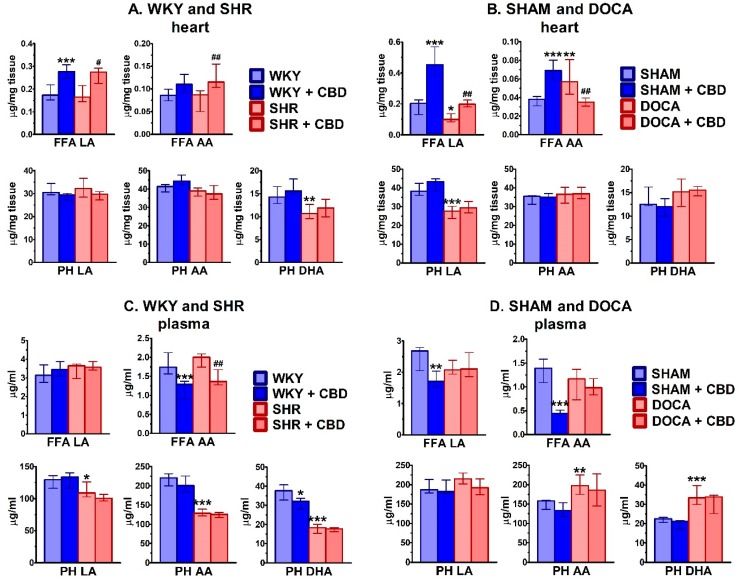
Influence of hypertension and cannabidiol (CBD) or its vehicle on levels of phospholipids (PH) and free fatty acids (FFA) in heart (**A**,**B**) and plasma (**C**,**D**) isolated from spontaneously (SHR; **A**,**C**) and deoxycorticosterone-salt (DOCA; **B**,**D**) hypertensive rats and their normotensive controls (WKY (**A**,**C**) and SHAM (**B**,**D**), respectively). CBD (10 mg/kg) was injected i.p. once daily for 14 days; the respective controls received the vehicle of CBD instead. Hearts were prepared and plasma was obtained 24 h after the final injection. Data are expressed as the median with interquartile range; *n* = 5–7; * *p* < 0.05; **/^##^ *p* < 0.01; *** *p* < 0.001 significantly different from WKY or SHAM (*) and from SHR or DOCA (#). LA—linoleic acid; AA—arachidonic acid; DHA—docosahexaenoic acid.

The effect of hypertension on free fatty acids and phospholipids was studied in cardiac tissue (Figure 7A,B) and plasma (Figure 7C,D). Among FFA, hypertension increased and decreased levels of AA (+50%) and of LA (−51%) in the heart of DOCA, respectively, but did not modify cardiac levels of FFA in SHR and plasma levels both in SHR and DOCA. In the fraction of phospholipids, hypertension increased plasma levels of AA (+25%) and DHA (+48%) and tended to increase plasma levels of LA in DOCA. On the other hand, it decreased cardiac levels of DHA (−25%) in SHR and LA (−28%) in DOCA and plasma levels of LA (−16%), AA (−42%) and DHA (−51%) in SHR.

Chronic CBD administration increased cardiac levels of FFA LA (+67%) and FFA AA (+32%) in SHR and FFA LA (+100%) in DOCA but decreased FFA AA in the heart of DOCA (−39%) and in the plasma of SHR (−32%). In normotensive controls, CBD increased or tended to increase cardiac levels of FFA LA (+60%) and FFA AA (+28%) in WKY and cardiac levels of FFA LA (+123%) and FFA AA (+82%) in SHAM. On the other hand, CBD decreased plasma levels of FFA AA (−26%) and PH DHA (−15%) in WKY and FFA LA (−36%) and FFA AA (-68%) in SHAM.

## 3. Discussion

### 3.1. General

This study shows that chronic CBD administration did not modify BP and HR in rats with primary (SHR) and secondary (DOCA-salt) hypertension in spite of the reduction of cardiac and plasma oxidative stress. It inhibited the FAAH activity in both hypertension models, but had opposite effects on cardiac levels of various endocannabinoids and endocannabinoid-related lipids (decrease in SHR vs. increase in DOCA). Unexpectedly, CBD increased lipid peroxidation in normotensive controls and this alteration may lead to untoward effects.

Our study is based on rats with primary (SHR; the most frequently studied genetic hypertensive model [17] and secondary hypertension (DOCA-salt [17]; because a salt-rich diet is one of the main lifestyle factors leading to hypertension). WKY and SHAM served as the respective normotensive controls and allowed us to detect potential undesirable effects of CBD. We used CBD at 10 mg/kg for 14 days since its chronic i.p administration had a beneficial effect on cardiovascular tissues (including decreases in oxidative stress) from diabetic (7 days; [50]) and septic rats (9 days; [28]) and from mice with diabetic (11 weeks; [22]) and doxorubicin-induced cardiomyopathy (5 days; [26]) and experimental autoimmune myocarditis (46 days; [51]). Note that CBD was used at ~ 10 mg/kg also in several acute experiments [10,13,15].

To examine the mechanism(s) of CBD action in hypertension, we considered parameters of oxidative stress (e.g., GSH and GSSG), of the endocannabinoid system (ECS) and of other components of lipid metabolism. With respect to the ECS, the two endocannabinoids AEA and 2-AG and the endocannabinoid-related lipids POEA, HEA, DEA, DGLEA, PEA, LEA, OEA, SEA and DHEA were determined [52]. The latter five and AEA are degraded by FAAH [53,54]. FAAH and MAGL activities and CB_1_, CB_2_, GPR18, GPR55 and TRPV1 receptor expression have been quantified as well. With respect to lipid metabolism free polyunsaturated fatty acids (PUFAs) including AA, LA and DHA and phospholipid PUFAs were determined.

The present results confirm and extend our previous observations [49] that in the heart, 2-AG has the highest concentration among the 11 endocannabinoids or endocannabinoid-related lipids. Its levels in WKY and SHAM were 220 and 75 times higher than the concentration of the best-known endocannabinoid AEA, respectively. This is an important observation in the light of recent publications that 2-AG in vivo worsens heart function after acute myocardial infarction [47], increases the severity of the cerebral blood flow deficit [55] or promotes atherogenesis [56] on the one hand but ameliorates inflammatory stress-induced insulin resistance in cardiomyocytes on the other [57]. Unfortunately, there are only limited data regarding the cardiovascular effects of endocannabinoids other than 2-AG and AEA. Thus, chronic administration of PEA (30 mg/kg for 5 weeks) or OEA (5 mg/kg for 7 days) improved rat myocardial function in doxorubicin-induced heart failure [58] and decreased BP and protected against kidney injury in SHR via inhibition of oxidative stress [59].

### 3.2. Effect of Hypertension

SBP was elevated in primary and secondary hypertension of animals in which BP was determined by the non-invasive (both models) and/or telemetric (SHR) method. With respect to SHR, both methods revealed an identical SBP (of about 170 mmHg) whereas HR differed. Compared to WKY, HR in SHR was higher or lower when the tail-cuff method or telemetry was used, respectively. A significant influence of restraint on HR determination by the non-invasive method (higher HR in SHR than in WKY) was described earlier [60]. In our previous study [49], DOCA-salt treatment over a period of 6 weeks resulted in a much higher SBP in DOCA than in SHR (about 220 vs. 185 mmHg, respectively). To avoid such a difference in the current study we reduced DOCA-salt treatment to 4 weeks (prior to the onset of the CBD treatment) which resulted in comparable SBP values on the final day of experiments in SHR and DOCA treated with vehicle (about 170 mmHg).

The elevated BP was connected with a redox imbalance, similarly to our previous papers [48,49]. Thus, GSSG increased and GSH decreased in heart and plasma of both hypertensive models. Other markers, including increased MDA, 4-HNE, 4-HHE and oxidative protein modifications (CO) occurred only in one hypertensive model and/or only in heart or plasma. In SHR (but not in DOCA) a decrease in plasma vitamin E and, contrarily to expectation, an increase in cardiac vitamin A was observed.

Cardiac FAAH activities increased in both models of hypertension like in our previous study [49]. Moreover, the present results show that in addition to AEA and 2-AG [49] also LEA, DHEA, POEA, HEA, DEA and DGLEA decreased (or tended to decrease) in the heart of SHR whereas in the heart of DOCA not only 2-AG but also LEA, OEA, DHEA, DEA and DGLEA increased. On the rule, changes in plasma endocannabinoid levels were similar (but less pronounced) with the exception that in DOCA OEA and DHEA levels decreased. Since numerous endocannabinoid-like compounds were changed in hypertensive animals, future studies should clarify whether these compounds affect the cardiovascular system. As suggested previously [49], the enhanced levels of AEA, LEA, OEA and SEA (all of which are degraded by FAAH) in spite of higher FAAH activity might indicate that in the heart of the DOCA group endocannabinoid synthesis is favoured [61] and/or AEA transporter activity is decreased [62].

Cardiac CB_1_ density decreased and GPR18 receptor density increased in both hypertension models whereas CB_2_ receptor density decreased in SHR only. The results obtained with cannabinoid and cannabinoid-like receptors only partially conform to those in our previous study [49]; differences may result from the fact that (i) receptor densities were determined in left ventricle vs. whole heart, respectively, and (ii) the time period for induction of DOCA-salt hypertension lasted for 4 vs. 6 weeks, respectively. As mentioned in the Introduction, stimulation of CB_1_ receptors enhances oxidative stress, whereas CB_2_ and GPR18 receptors have an opposite effect. Accordingly, the hypertension-induced enhancement in oxidative stress does not fit well to the changes in receptor densities since in both hypertension models, a decreased pro-oxidative receptor (CB_1_) was connected with an increased anti-oxidative receptor (GPR18).

The hypertension-related changes in lipids other than the endocannabinoids were dependent on the hypertension model. Similarly to endocannabinoids, mainly decreases in phospholipids (cardiac PH DHA and plasma PH LA, AA and DHA) were obtained in SHR. By contrast, in DOCA plasma phospholipid AA and DHA and cardiac FFA AA increased whereas cardiac FFA LA and PH LA decreased. Differences in particular cardiac and plasma PUFAs between SHR and DOCA were also observed in our previous study [49].

### 3.3. Effect of Chronic CBD in Hypertensive Animals

Chronic administration of CBD failed to modify BP and HR in both models of hypertension; this is in harmony with our previous study [15], in which a single CBD administration had no effect. One may argue that the dose of CBD was too low or the duration of its application too short; however, both parameters conformed to the conditions chosen in previous studies in which CBD had a beneficial effect (see 3.1). One may also argue that the effectiveness of CBD was lost during the study; however, FAAH (which is inhibited by CBD; [43]) was still reduced (DOCA) or tended to be reduced (SHR) 24 h after the final dose of CBD.

How can we explain the lack of an antihypertensive effect of CBD in hypertension? CBD does not only possess vasodilatory activity mainly shown on isolated vessels (for review, see [9]) but, according to our recent study in pithed SHR and WKY [15], at an intravenous dose of 10 mg/kg, it also exhibits sympathomimetic effects. Opposite cardiovascular effects might be at least partially responsible for the lack of a hypotensive effect of CBD in our study.

Hypertension is associated with an enhancement of oxidative stress ([16]; current study) and CBD is known for its antioxidant properties (for literature, see Introduction). Indeed, chronic CBD administration led to increases in GSH and decreases in GSSG both in heart and plasma in both hypertension models. Moreover, CBD counteracted parameters of lipid peroxidation including the enhanced cardiac and/or plasma 4-HNE and 4-HHE in SHR and the enhanced cardiac and plasma MDA and 4-HNE in DOCA. Moreover, CBD reduced the enhanced concentration of carbonyl groups in plasma. The increase in plasma vitamin E in DOCA was another beneficial effect of CBD although, contrarily to expectation, cardiac levels of vitamin A and E in SHR decreased.

The endocannabinoid system is overactivated in hypertension [63]. Chronic CBD administration failed to modify the densities of cannabinoid and cannabinoid-like receptors (the only exception was the decrease in cardiac GPR55 levels in SHR) but decreased cardiac levels of 2-AG, OEA, DEA and DGLEA in DOCA and plasma levels of AEA and LEA in DOCA and of SEA, HEA and DGLEA in SHR. On the rule, CBD reduced the levels of those ECBs the concentrations of which were enhanced in hypertension. Accordingly, CBD failed to modify cardiac endocannabinoid levels in SHR since only decreases were obtained. Surprisingly, the decreases in ECBs were observed in spite of the reduced FAAH activity. Due to the vasodilatory and/or hypotensive effects of AEA and OEA [64,65], the CBD-induced decrease in AEA and OEA levels appears to be unfavourable in hypertension whereas the decrease in cardiac 2-AG concentration might be beneficial because worsening of cardiac function by 2-AG has been described recently [47].

The effect of CBD on PUFAs was again dependent on the hypertension model and on the level of PUFAs in hypertension. Hypertension-induced increases in PUFAs were observed in DOCA only and CBD decreased the cardiac FFA AA and tended to decrease the plasma PH AA and DHA.

### 3.4. Effect of Chronic CBD in Normotensive Animals

CBD is generally recognized as a safe drug [66]. Surprisingly, we have obtained some unexpected effects of chronic CBD administration in normotensive rats. The most untoward influence was the enhancement of lipid peroxidation documented by increases of heart and plasma MDA, 4-HNE and 4-HHE in SHAM and, in addition, of plasma 4-HHE in WKY. These alterations were connected with a decrease in cardiac (antioxidant) GPR18 receptor density in WKY and an increase (or the tendency of an increase) in cardiac FFA LA and FFA AA in WKY and SHAM. Our findings, although not unequivocal (since in plasma decreases in FFA AA (WKY and SHAM), FFA LA (SHAM) and HEA (WKY) occurred), are reminiscent of a previous study [28], in which CBD (10 mg/kg) administered to rats for 9 days led to an increase in carbonyl groups in lung and liver.

### 3.5. Limitations of the Study

The present investigation, in which a blood pressure-lowering effect of CBD could not be shown despite its antioxidant effect, was restricted to two models of hypertension studied in male rats. The possibility has to be considered that an antihypertensive effect of CBD was missing for the following reasons. (1) The duration of CBD administration might have been too short or its dose too low and an increase in the duration of its application or the use of a higher dose might have led to more evident changes, especially of the markers of oxidative stress and probably also of the level of blood pressure. (2) In our previous paper, the chronic administration of the FAAH inhibitor URB597 decreased BP in DOCA but not in SHR, in which BP before the first dose of URB597 was about 220 vs. 185 mmHg, respectively [67]. As mentioned above, in the present study, the period for the induction of DOCA hypertension was reduced from 6 to 4 weeks, which resulted in lower SBP (about 170 mmHg). We cannot exclude that the lack of effect of CBD is related to the shorter induction time and the lower blood pressure level in DOCA. (3) We used DOCA as a secondary model of hypertension. The possibility has to be considered that a model of hypertension connected with changes in the renin-angiotensin-aldosterone system (RAAS) (the activity of which is modified by the endocannabinoid system) might have revealed a blood pressure-lowering effect of CBD. Interestingly, opposite effects of a CB_1_ receptor antagonist have been found in the cardiovascular system of SHR and of rats with a RAAS-dependent hypertension (for review, see [63]). (4) There are gender-specific differences in hypertension [17,68] and it would be interesting to extend our experiments to female rats. (5) There is no ideal animal model of human hypertension. However, the advantage of rat genetic models of hypertension is their similarity to the BP/hypertension phenotypes observed in patients and that SHR responds to the antihypertensive effects of almost all classes of drugs approved for treatment of hypertension. The DOCA-salt model connected with unilateral nephrectomy provides a reliable animal model that can develop severe hypertension with some features of human low-renin hypertension [17].

The mechanism of action for the antioxidant effect of CBD was not determined. This is not a trivial task since the compound has many different molecular effects [1,3,4,29]. The fact that CBD influenced components of the ECS in the two hypertension models in an opposite manner suggests that a direct antioxidant effect is more likely than an indirect ECS-based mechanism. Finally, although the antioxidant effect of CBD was not associated with an antihypertensive effect it would be interesting to examine the influence of CBD on other cardiovascular parameters, e.g., arrhythmia risk, in future studies. Thus, oxidative stress was shown to be involved in cardiac electrical and structural remodelling [69] and in the pathophysiology of atrial fibrillation [70]. The use of ECG predictors of arrhythmia risk would be a benefit in such studies [69,71].

## 4. Materials and Methods

### 4.1. Animals

All procedures and experimental protocols were performed in accordance with the European Directive (2010/63/EU) and with the approval of the local Animal Ethics Committee in Olsztyn (Poland) (Approval code: 80/2017; approval date: 28 November 2017). Rats were obtained from the Center for Experimental Medicine of the Medical University of Białystok (Poland). They had free access to chow and water and were kept under a 12:12 h light-dark cycle. Experiments were performed on male rats with spontaneous (SHR) and secondary DOCA-salt hypertension.

### 4.2. Experimental Groups and Protocol

DOCA-salt hypertension was induced in Wistar rats. After unilateral nephrectomy of 5-6 week-old animals and one week of recovery, deoxycorticosterone acetate (DOCA) was injected s.c. (25 mg/kg in 0.4 mL DMF/kg) twice a week for 28 days. During DOCA administration, drinking water was replaced with 1% saline water. Control group for DOCA were sham-operated (unilaterally nephrectomised) (SHAM) Wistar rats. They received s.c. DMF (DOCA vehicle) twice weekly for 4 weeks and tap water for drinking.

Animals were randomly divided into experimental groups: (1) hypertensive DOCA rats, (2) respective normotensive control SHAM rats, (3) hypertensive SHR rats and (4) respective normotensive WKY rats. All animal groups were age-matched at the beginning of CBD treatment (8–9 weeks old). The body weight of the rats is shown in Table 1.

### 4.3. Chronic CBD Administration

One part of every hypertensive and normotensive group were injected i.p. with CBD (10 mg/kg) every 24 h for 14 days. The other part received CBD vehicle (ethanol, Tween 80, 0.9% NaCl—3:1:16; 1 mL/kg).

### 4.4. Determination of Cardiovascular Parameters in Conscious Rats

Systolic blood pressure (SBP) and heart rate (HR) were measured using non-invasive tail-cuff method with Non-Invasive Blood Pressure Controller (ADInstruments, Sydney, Australia) before first dose of CBD or its vehicle and after 7 and 14 days of experiment (24 h after last dose of compounds).

SBP, diastolic blood pressure (DBP) and HR were also measured telemetrically in SHR and WKY rats, as described previously [15]. Briefly, after pentobarbitone sodium (300 μmol/kg; i.e., ~70 mg/kg; i.p.) anesthesia, telemetry transmitters (HD-S10, Data Sciences International, Saint Paul, MN, USA) were implanted into the femoral artery. Rats were allowed to recover for 1 week before measurements.

### 4.5. Tissue Preparation for Biochemical Examinations

Twenty-four hours after the last dose of CBD or its vehicle rats were anesthetized with pentobarbitone sodium (300 μmoL/kg; i.p.) to collect blood and heart. Blood samples were obtained by left ventricle puncture and collected into EDTA tubes. Plasma separation from whole blood was carried out by centrifugation at 2000× *g* for 5 min.

Hearts were perfused with 0.9% saline and cut lengthwise into two halves with equal size and quality. The first part of the tissue was snap-frozen with liquid nitrogen and stored at −80 °C. The second part was homogenized. 10% homogenates in 0.9% saline were centrifuged at 20000× *g* for 15 min at 4 °C.

### 4.6. Biochemical Studies

#### 4.6.1. Determination of Endocannabinoids

Anandamide (AEA), 2-arachidonoylglycerol (2-AG), palmitoyl ethanolamide (PEA), linolenoyl ethanolamide (LEA), oleoyl ethanolamide (OEA), stearoyl ethanolamide (SEA), docosahexaenoyl ethanolamide (DHEA), palmitoleoyl ethanolamide (POEA), homo-γ-linolenyl ethanolamide (HEA), docosatetraenoyl ethanolamide (DEA) and dihomo-γ-linolenoyl ethanolamide (DGLEA) were determined using modified ultrahigh performance liquid chromatography-tandem mass spectrometry (UPL-CMS/MS) by the Lam method [72]. Octadeuterated endocannabinoids: AEA-d_8_, 2-AG-d_8_ and OEA-d_4_ [73] as internal standards were added into the tissue lysates and all cannabinoids were isolated using a solid phase extraction (OASIS HLB 3cc). UPLC–MS/MS analysis was carried out using an Nexera X2 Shimadzu UPLC system with a Zorbax Extend C18 column (2.1 mm× 150 mm, 1.8 mm, Agilent, Santa Clara, CA, USA) and interfaced with a Shimadzu 8060 triple quadrupole mass spectrometer with electrospray ionization source (ESI). The samples were analyzed in positive-ion mode using multiple reaction monitoring (MRM). Transitions of the precursor to the product ion was as follows: m/z 348.3→62.15 for AEA, m/z 379.3→287.25 for 2-AG, m/z 300.3→62.00 for PEA, m/z 324.0→62.00 for LEA, m/z 326.0→62.00 for OEA, m/z 328.0→62.00 for SEA, m/z 372.0→62.00 for DHEA, m/z 298.0→62.00 for POEA, m/z 314.5→62.00 for HEA, m/z 376.0→62.00 for DEA and m/z 350.0→62.00 for DGLEA.

#### 4.6.2. Determination of FAAH and MAGL Activity

Fatty acid amide hydrolase (FAAH) (EC.3.5.1.99) activity was measured in the homogenate of heart tissue prepared in 20 mM Tris, containing 10% glycerol, 150 mM NaCl, and 1% Triton X-100, pH 7.8 at 4 °C. Following centrifugation (1000× *g*), 20 μL of the supernatant was added to 175 μL of reaction buffer (125 mM Tris, pH 9.0, and 1 mM EDTA) and 17 μM of FAAH substrate, decanoyl m-nitroaniline. Formation of m-nitroaniline (m-NA) was determined at 410 nM [74]. Specific enzyme activity was expressed in nmoles of m-NA/min/mg protein.

Monoacylglycerol lipase (MAGL) (EC.3.1.1.23) activity was measured in the homogenate of heart tissue prepared in 20 mM Tris, 320 mM sucrose and 1mM EDTA, pH 8.0. Heart supernatant was obtained from the soluble fraction after spinning the homogenate at 1000× *g* for 15 min. A reaction mixture containing heart supernatants, 10 mM Tris, 1 mM EDTA pH 7.2 was pre-incubated at 4 °C for 15 min. After addition of arachidonoyl-1-thio-glycerol (A-1-TG), the mixture was incubated at 37 °C for 5 min and after refrigerating to room temperature, 1 mM DTNB was added. After 3 min, the formation of TNB was determined at 412 nm. Specific enzyme activity was expressed in nmoles of TNB/min/mg of protein [75].

#### 4.6.3. Western Blot Analysis

A routine Western blotting procedure was used to examine protein expression, as has been described previously [67], except using stain-free technology [76]. Briefly, samples from the left ventricles were homogenized in radioimmunoprecipitation assay (RIPA) buffer containing a cocktail of protease and phosphatase inhibitors. In addition, protein concentration was measured using the bicinchoninic acid method (BCA) with bovine serum albumin (BSA) as a standard. Subsequently, homogenates were reconstituted in Laemmli buffer. The same amounts of protein (30 µg) were loaded on Criterion™ TGX Stain-Free Precast Gels (Bio-Rad, Hercules, CA, USA). After electrophoresis, proteins were transferred onto PVDF (polyvinylidene difluoride) membranes using Trans-Blot Turbo Transfer System (Bio-Rad, Hercules, CA, USA) and stain-free blot image was taken (ChemiDoc XRS; Bio-Rad, Hercules, CA, USA) for total protein measurement in each sample lane. Next, the membranes were incubated overnight at 4 °C with the corresponding primary antibodies in appropriate dilutions: CB_1_ (1:500), CB_2_ (1:500), GPR18 (1:5000), GPR55 (1:1000) and TRPV1 (1:500). Thereafter, PVDF membranes were incubated with the appropriate secondary antibody conjugated to horseradish peroxidase (Cell Signaling Technology, Danvers, MA, USA). After adding a suitable substrate for horseradish peroxidase protein bands were detected using a ChemiDoc visualization system XRS (Bio-Rad, Hercules, CA, USA). Thereafter, Western blots were quantified densitometrically with Image Laboratory Software Version 6.0.1 (Bio-Rad, Hercules, CA, USA). The expression of selected target proteins was quantified using stain-free gels and total protein normalization method (Bio-Rad, Hercules, CA, USA).

#### 4.6.4. Determination of Antioxidant Enzyme Activity

Catalase (CAT—EC.1.11.1.9) activity was measured in the homogenate of heart tissue by a spectrophotometric analysis (at 240 nm) of the rate of hydrogen peroxide decomposition, using a method published previously [77]. One unit of CAT is defined as the amount of the enzyme necessary to catalyze the decomposition of 1 µmoL of hydrogen peroxide to water and oxygen within 1 min.

Glutathione reductase (GSR—EC.1.6.4.2) activity was measured according to the method of Mize and Langdon [78] by monitoring the oxidation of NADPH at 340 nm at a pH 7.4. One unit of GSR oxidized 1 µmol of NADPH/min at 25 °C and pH 7.4. Specific enzyme activity was expressed in units per mg of protein.

Glutathione peroxidase (GPx—EC.1.11.1.9) activity was assessed spectrophotometrically using the method of Paglia and Valentine [79]. GPx activity was assayed by measuring the conversion of NADPH to NADP^+^. One unit of GPx activity was defined as the amount of enzyme catalyzing the oxidation of 1 μmoL NADPH/min at 25 °C and pH 7.4. Specific enzyme activity was expressed in units per mg of protein.

Superoxide dismutase (SOD—EC.1.15.1.1) activity was measured according the method by Sykes et al. [80]. The oxidation of epinephrine was performed in terms of the production of adrenochrome, which has a maximal absorption at 480 nm. One unit of SOD is defined as the amount of the enzyme that inhibits the rate of autoxidation of epinephrine by 50%.

#### 4.6.5. Determination of Non-Enzymatic Antioxidant Level

Vitamin E and A were detected in the samples using high-performance liquid chromatography (HPLC) [81]. Extraction of vitamins was carried out using hexane. After removal, drying and dilution with ethanol, the hexane phase (50 μL) was injected on the column. UV detection at 294 nm for vitamin E and 298 nm for vitamin A were applied. The flow rate was 1 mL/min of methanol and water (95:5).

Glutathione (GSH) and glutathione disulfide (GSSG) were quantified using the capillary electrophoresis (CE) method of Maeso et al. [82]. Samples were sonificated in the Eppendorf tubes with 2 mL of a mixture containing ACN/H_2_O (62.5:37.5, *v*/*v*) and centrifuged at 29,620× *g* for 10 min. The supernatant was immediately measured by CE. The separation was performed on a capillary with 47 cm total length (40 cm effective length) and 50 μm ID and was operated at 27 kV with UV detection at 200 ± 10 nm.

#### 4.6.6. Determination of Protein Modifications

Protein oxidative modifications (carbonyl groups; CO groups) were determined according to the method published previously [83]. Carbonyl content was computed from peak absorption (370 nm) using 2,4-dinitrophenylhydrazine as a reagent.

#### 4.6.7. Determination of Lipid Modifications

Malondialdehyde (MDA), 4-hydroxynonenal (4-HNE) and 4-hydroxyhexenal (4-HHE) were measured by GC/MSMS, as the O-PFBoxime-TMS derivatives, using modified method of Luo et al. [84]. Benzaldehyde-D6 as an internal standard was added to the tissue lysates and aldehydes were derivatized by the addition of O-(2,3,4,5,6-pentafluorobenzyl) hydroxyamine hydrochloride (0.05 M in PIPES buffer, 200 μL; incubation for 60 min at room temperature). Subsequently, samples were deproteinized by the addition of 1 mL of methanol and OPFB-oxime aldehyde derivatives were extracted by the addition of 2 mL of hexane. The top hexane layer was transferred into borosilicate tubes and evaporated under a stream of argon gas followed by the addition of N,O-bis(trimethylsilyl)trifluoroacetamide in 1% trimethylchlorosilane. A 1 μL aliquot was injected on the column. Derivatized aldehydes were analyzed using a 7890A GC—7000 quadrupole MS/MS (Agilent Technologies, Palo Alto, CA) equipped with a HP-5 ms capillary column (0.25 mm internal diameter, 0.25 μm film thickness, 30 m length). Derivatized aldehydes were detected in the selected ion monitoring (SIM) mode. The ions used for MDA/4-HNE/4HHE-PFB-TMS identification were m/z 204.0 and 178.0 for MDA; m/z 333.0 and 181.0 for 4-HNE; and 352.0 and 226.0 for 4-HHE respectively and m/z 307.0 for IS (benzaldehyde-D6) derivatives.

#### 4.6.8. Determination of Fatty Acids

The concentration of the fatty acids AA, DHA and LA was determined by gas chromatography [85]. Lipid components were isolated from tissue lysates by extraction with chloroform/methanol mixture (2:1, v/v). Using TLC, total phospholipids were separated with the mobile phase heptane—diisopropyl ether—acetic acid (60:40:3, *v*/*v*/*v*). All lipid fractions were transmethylated to fatty acid methyl esters (FAMEs) with boron trifluoride in methanol reagent under nitrogen atmosphere without previous separation from the layer. The FAMEs were quantified by gas chromatography with a flame ionization detector. Separation of FAME was carried out on a capillary column coated with Varian CP-Sil88 stationary phase and analyzed by gas chromatography with a flame ionization detector (FID) on a Clarus 500 Gas Chromatograph (Perkin Elmer, Waltham, MA, USA).

### 4.7. Statistical Analysis

The results are expressed as median values and interquartile range. Cardiovascular parameters were obtained from WKY and SHR rats in which blood pressure was recorded telemetrically (*n* = 4). Rats, in which blood pressure was determined by the tail-cuff method, served both for the registration of cardiovascular and biochemical parameters (WKY, SHR, SHAM, DOCA-salt). At the beginning, each group consisted of 7 rats. However, the final *n* was 5-7 because of (1) the death of one rat (DOCA + CBD) and/or (2) the exclusion of outliers (values deviating from the mean by more than plus/minus three standard deviations). All data were subjected to the Kolmogorov–Smirnov test to assess the distribution of values. If the data were normally distributed, parametric tests were done (paired Student’s t-test for comparison within group and one-way ANOVA with Bonferroni’s multiple comparison test for multiple groups). Data subjected to ANOVA were followed by Bonferroni’s post hoc tests only when the F value attained *p* < 0.05 and there was no significant inhomogeneity of variances. If the data were not normally distributed, a non-parametric test was performed (Wilcoxon test for comparison within group and Kruskal–Wallis test with Dunn’s post hoc test to compare multiple groups). Dunn’s post hoc test was only used when the Kruskal–Wallis test yielded a significant result (*p* < 0.05). A statistical analysis was performed using Graph Pad Prism 5 (GraphPad Software, La Jolla, CA, USA).

### 4.8. Drugs

(-)-cannabidiol (CBD) (THC-1073G-1) from THC Pharm, Frankfurt, Germany; ethanol (BA6420113) and natrium chloride (NaCl) (BA4121116) from POCH, Gliwice, Poland; Tween 80 (P1754), 11-deoxycorticosterone acetate (DOCA) (D7000), N,N-dimethylformamide (DMF) (9227056), chloro-2,4-dinitro benzene (CDNB) (237329), butylated hydroxytoluene (BHT) (W218405) and 5,5′-dithiobis(2-nitrobenzoic acid) (DTNB) (D8130) from Sigma-Aldrich, Munich, Germany; pentobarbital sodium (5909991290153) from Biowet, Puławy, Poland; anandamide-d8 (AEA-d8) (390050), 2-arachidonoyl glycerol-d_8_ (2-AG-d_8_) (362160), oleoyl ethanolamide-d_4_ (OEA-d_4_) (9000552), decanoyl m-nitroaniline (m-NA) (90349), arachidonoyl-1-thio-glycerol (A-1-TG) (10007904) from Cayman Chemical Company, Ann Arbor, MI, USA; CB_1_ antibody (ab23703), CB_2_ antibody (ab3561), GPR18 antibody (ab174835), GPR55 antibody (ab203663) from Abcam, Cambridge, UK; TRPV1 antibody (bs-1931R) from Bioss Antibodies, Woburn, MA, USA; Clarity Western ECL Substrate (1705060) from Bio-Rad, Hercules, CA, USA.

## 5. Conclusions

Chronic CBD administration (10 mg/kg once a day for two weeks) does not modify BP and HR in a model of primary (SHR) and secondary (DOCA-salt) hypertension and in their respective normotensive controls in spite of the reduction of cardiac and plasma oxidative stress. Whether, besides its direct effect, CBD also possesses an indirect anti-oxidant effect that is based on the endocannabinoid system is questionable. Thus, CBD had opposite effects on numerous components of the endocannabinoid system in both hypertension models. The unexpected CBD-related increase in lipid peroxidation in normotensive controls deserves further investigation and may lead to untoward effects if CBD is used for therapeutical purposes listed in the Introduction. Provided that our data on animals can be transferred to humans, CBD will not lead to an unexpected fall in blood pressure in patients.

## Figures and Tables

**Table 1:** Influence of cannabidiol (CBD) or its vehicle on systolic blood pressure, heart rate and body weight in conscious spontaneously (SHR) and deoxycorticosterone (DOCA) hypertensive rats and their respective normotensive controls (Wistar-Kyoto (WKY) and sham-operated (SHAM) rats).

Group	Treatment	*n*	Systolic Blood Pressure (mmHg)		Heart Rate (Beats/min)		Body Weight (g)	
			Before the First Dose of CBD or Its Solvent	24 h After the Final Dose of CBD or Its Solvent	Before the First Dose of CBD or Its Solvent	24 h After The Final Dose of CBD or Its Solvent	Before the First Dose of CBD or Its Solvent	24 h After the Final Dose of CBD or Its Solvent
WKY	vehicle	7	111 [95;122]	98 [91;105] ^!^	341 [321;366]	314 [304;326]	342 [329;361]	380 [367;400] ^!!!^
WKY	CBD	7	101 [87;124]	92 [87;107]	334 [313;357]	300 [298;321] ^!^	325 [325;340]	360 [353;390] ^!^
SHR	vehicle	7	178 [161;199] ***	174 [164;187] ***	359 [351;369]	367 [365;387] **	290 [282;292] ***	300 [296;326] ***^!^
SHR	CBD	7	184 [172;192] ^$$$^	172 [170;176] ^$$$^	355 [352;397]	358 [344;380] ^$$^	315 [300;325]	328 [313;340] ^$!!!^
SHAM	vehicle	7	122 [111;132]	120 [118;131]	363 [351;371]	336 [331;340] ^!^	294 [270;304]	320 [302;345] ^!!!^
SHAM	CBD	7	111 [100;129]	121 [109;134]	354 [333;374]	349 [328;371]	278 [256;294]	322 [286;340] ^!^
DOCA	vehicle	7	163 [111;175]	175 [160;190] ***	314 [303;360]	324 [312;352]	275 [233;276]	280 [252;321]
DOCA	CBD	6	150 [143;174] ^$^	173 [150;189] ^$$$^	327 [315;354]	314 [301;348]	280 [271;298]	303 [297;312] ^!^

Cardiovascular parameters were measured by the non-invasive tail-cuff method. CBD (10 mg/kg) or its vehicle were injected intraperitoneally once daily for 14 days. Data are expressed as the medians with interquartile range; ^$^/^!^ *p*< 0.05; **/^$$^ *p* < 0.01; ***/^$$$^/^!!!^ *p* < 0.001 significantly different from the respective values obtained in normotensive groups receiving vehicle for CBD (*) or CBD (^$^) or recorded before the first dose of the compound (^!^).

## Abbreviations

2-AG	2-arachidonoylglycerol
4-HHE	4-hydroxyhexenal
4-HNE	4-hydroxynonenal
AA	arachidonic acid
ACN	acetonitrile
AEA	anandamide
A-1-TG	arachidonoyl-1-thio-glycerol
ANOVA	analysis of variance
BCA	bicinchoninic acid
BP	blood pressure
BSA	bovine serum albumin
CAT	catalase
CBD	cannabidiol
CE	capillary electrophoresis
CO	carbonyl groups
DBP	diastolic blood pressure
DEA	docosatetraenoyl ethanolamide
DGLEA	dihomo-γ-linolenoyl ethanolamide
DHA	docosahexaenoic acid
DHEA	docosahexaenoyl ethanolamide
DMF	dimethylformamide
DOCA	deoxycorticosterone acetate *or* DOCA-salt hypertensive rats
DOCA-salt	DOCA-based method to generate hypertension in rats *or* DOCA-salt hypertensive rats
DTNB	5,5′-dithiobis-2-dinitrobenzoic acid
ECG	electrocardiography
ECS	endocannabinoid system
EDTA	ethylenediaminetetraacetic acid
ESI	electrospray ionization source
FAAH	fatty acid amide hydrolase
FAME	fatty acid methyl esters
FFA	free fatty acids
FID	flame ionization detector
GC	gas chromatography
GPR	G-protein coupled receptor
GPx	glutathione peroxidase
GSH	glutathione
GSR	glutathione-disulfide reductase
GSSG	glutathione disulfide
HEA	homo-γ-linolenyl ethanolamide
HPLC	high-performance liquid chromatography
HR	heart rate
ID	internal diameter
i.p.	intraperitoneal injection
IS	internal standard
LA	linoleic acid
LEA	linolenoyl ethanolamide
MAGL	monoacylglycerol lipase
MDA	malondialdehyde
MRM	multiple reaction monitoring
MS/MS	tandem mass spectrometry
m-NA	m-nitroaniline
NADA	N-arachidonoyl dopamine
NADPH	nicotinamide adenine dinucleotide phosphate
OEA	oleoyl ethanolamide
O-PFBoxime	O-(2,3,4,5,6-pentafluorobenzyl) oxime
PEA	palmitoyl ethanolamide
PH	phospholipids
PIPES	piperazine-N,N′-bis(2-ethanesulfonic acid)
POEA	palmitoleoyl ethanolamide
PPR	peroxisome proliferator-activated receptor
PUFA	polyunsaturated fatty acids
PVDF	polyvinylidene difluoride
RAAS	renin-angiotensin-aldosterone system
RIPA	radioimmunoprecipitation assay
SBP	systolic blood pressure
SEA	stearoyl ethanolamide
SEM	standard error of the mean
SHAM	sham-operated rats
SHR	spontaneously hypertensive rats
SIM	selected ion monitoring mode
SOD	superoxide dismutase
s.c.	subcutaneous injection
THC	Δ^9^-tetrahydrocannabinol
TLC	thin-layer chromatography
TNB	5-thio-2-nitrobenzoic acid
TRPV	transient receptor potential vanilloid
UPLC	ultrahigh performance liquid chromatography
WKY	Wistar-Kyoto rats

## References

[1] Pisanti S., Malfitano A.M., Ciaglia E., Lamberti A., Ranieri R., Cuomo G., Abate M., Faggiana G., Proto M.C., Fiore D. (2017). Cannabidiol: State of the art and new challenges for therapeutic applications. Pharmacol. Ther..

[2] White C.M. (2019). A review of human studies assessing cannabidiol’s (CBD) therapeutic actions and potential. J. Clin. Pharmacol..

[3] Booz G.W. (2011). Cannabidiol as an emergent therapeutic strategy for lessening the impact of inflammation on oxidative stress. Free Radic. Biol. Med..

[4] Millar S.A., Stone N.L., Bellman Z.D., Yates A.S., England T.J., O’Sullivan S.E. (2019). A systematic review of cannabidiol dosing in clinical populations. Br. J. Clin. Pharmacol..

[5] Gruden G., Barutta F., Kunos G., Pacher P. (2016). Role of the endocannabinoid system in diabetes and diabetic complications. Br. J. Pharmacol..

[6] Paloczi J., Varga Z.V., Haskó G., Pacher P. (2018). Neuroprotection in oxidative stress-related neurodegenerative diseases: Role of endocannabinoid system modulation. Antioxid. Redox Signal..

[7] Atalay S., Jarocka-Karpowicz I., Skrzydlewska E. (2019). Antioxidative and anti-inflammatory properties of cannabidiol. Antioxidants.

[8] Franco V., Perucca E. (2019). Pharmacological and therapeutic properties of cannabidiol for epilepsy. Drugs.

[9] Stanley C.P., Hind W.H., O’Sullivan S.E. (2013). Is the cardiovascular system a therapeutic target for cannabidiol?. Br. J. Clin. Pharmacol..

[10] Jadoon K.A., Tan G.D., O’Sullivan S.E. (2017). A single dose of cannabidiol reduces blood pressure in healthy volunteers in a randomized crossover study. JCI Insight.

[11] Sultan S.R., Millar S.A., England T.J., O’Sullivan S.E. (2017). A systematic review and meta-analysis of the haemodynamic effects of cannabidiol. Front. Pharmacol..

[12] Sultan S.R., England T.J., O’Sullivan S.E. Acute and chronic effects of cannabidiol on haemodynamics in healthy males. Proceedings of the 29th Annual Symposium on the Cannabinoids.

[13] Resstel L.B., Tavares R.F., Lisboa S.F., Joca S.R., Corrêa F.M., Guimarães F.S. (2009). 5-HT_1A_ receptors are involved in the cannabidiol-induced attenuation of behavioural and cardiovascular responses to acute restraint stress in rats. Br. J. Pharmacol..

[14] Granjeiro E.M., Gomes F.V., Guimarães F.S., Corrêa F.M., Resstel L.B. (2011). Effects of intracisternal administration of cannabidiol on the cardiovascular and behavioral responses to acute restraint stress. Pharmacol. Biochem. Behav..

[15] Kossakowski R., Schlicker E., Toczek M., Weresa J., Malinowska B. (2019). Cannabidiol affects the Bezold-Jarisch reflex via TRPV1 and 5-HT_3_ receptors and has peripheral sympathomimetic effects in spontaneously hypertensive and normotensive rats. Front. Pharmacol..

[16] Knock G.A. (2019). NADPH oxidase in the vasculature: Expression, regulation and signalling pathways; role in normal cardiovascular physiology and its dysregulation in hypertension. Free Radic. Biol. Med..

[17] Lerman L.O., Kurtz T.W., Touyz R.M., Ellison D.H., Chade A.R., Crowley S.D., Mattson D.L., Mullins J.J., Osborn J., Eirin A. (2019). Animal models of hypertension: A scientific statement from the American Heart Association. Hypertension.

[18] Baranowska-Kuczko M., Kozłowska H., Kloza M., Sadowska O., Kozłowski M., Kusaczuk M., Kasacka I., Malinowska B. (2019). Vasodilatory effects of cannabidiol in human pulmonary and rat small mesenteric arteries: Modification by hypertension and the potential pharmacological opportunities. J. Hypertens..

[19] Rajesh M., Mukhopadhyay P., Bátkai S., Haskó G., Liaudet L., Drel V.R., Obrosova I.G., Pacher P. (2007). Cannabidiol attenuates high glucose-induced endothelial cell inflammatory response and barrier disruption. Am. J. Physiol. Heart Circ. Physiol..

[20] Mukhopadhyay P., Rajesh M., Horváth B., Bátkai S., Park O., Tanchian G., Gao R.Y., Patel V., Wink D.A., Liaudet L. (2011). Cannabidiol protects against hepatic ischemia/reperfusion injury by attenuating inflammatory signaling and response, oxidative/nitrative stress, and cell death. Free Radic. Biol. Med..

[21] Sun S., Hu F., Wu J., Zhang S. (2017). Cannabidiol attenuates OGD/R-induced damage by enhancing mitochondrial bioenergetics and modulating glucose metabolism via pentose-phosphate pathway in hippocampal neurons. Redox Biol..

[22] Rajesh M., Mukhopadhyay P., Bátkai S., Patel V., Saito K., Matsumoto S., Kashiwaya Y., Horváth B., Mukhopadhyay B., Becker L. (2010). Cannabidiol attenuates cardiac dysfunction, oxidative stress, fibrosis, and inflammatory and cell death signaling pathways in diabetic cardiomyopathy. J. Am. Coll. Cardiol..

[23] El-Remessy A.B., Al-Shabrawey M., Khalifa Y., Tsai N.T., Caldwell R.B., Liou G.I. (2006). Neuroprotective and blood-retinal barrier-preserving effects of cannabidiol in experimental diabetes. Am. J. Pathol..

[24] Yang L., Rozenfeld R., Wu D., Devi L.A., Zhang Z., Cederbaum A. (2014). Cannabidiol protects liver from binge alcohol-induced steatosis by mechanisms including inhibition of oxidative stress and increase in autophagy. Free Radic. Biol. Med..

[25] Wang Y., Mukhopadhyay P., Cao Z., Wang H., Feng D., Haskó G., Mechoulam R., Gao B., Pacher P. (2017). Cannabidiol attenuates alcohol-induced liver steatosis, metabolic dysregulation, inflammation and neutrophil-mediated injury. Sci. Rep..

[26] Hao E., Mukhopadhyay P., Cao Z., Erdélyi K., Holovac E., Liaudet L., Lee W.S., Haskó G., Mechoulam R., Pacher P. (2015). Cannabidiol protects against doxorubicin-induced cardiomyopathy by modulating mitochondrial function and biogenesis. Mol. Med..

[27] Fouad A.A., Albuali W.H., Al-Mulhim A.S., Jresat I. (2013). Cardioprotective effect of cannabidiol in rats exposed to doxorubicin toxicity. Environ. Toxicol. Pharmacol..

[28] Cassol O.J., Comim C.M., Silva B.R., Hermani F.V., Constantino L.S., Felisberto F., Petronilho F., Hallak J.E., De Martinis B.S., Zuardi A.W. (2010). Treatment with cannabidiol reverses oxidative stress parameters, cognitive impairment and mortality in rats submitted to sepsis by cecal ligation and puncture. Brain Res..

[29] Kis B., Ifrim F.C., Buda V., Avram S., Pavel I.Z., Antal D., Paunescu V., Dehelean C.A., Ardelean F., Diaconeasa Z. (2019). Cannabidiol-from plant to human body: A promising bioactive molecule with multi-target effects in cancer. Int. J. Mol. Sci..

[30] Lipina C., Hundal H.S. (2016). Modulation of cellular redox homeostasis by the endocannabinoid system. Open Biol..

[31] Gallelli C.A., Calcagnini S., Romano A., Koczwara J.B., de Ceglia M., Dante D., Villani R., Giudetti A.M., Cassano T., Gaetani S. (2018). Modulation of the oxidative stress and lipid peroxidation by endocannabinoids and their lipid analogues. Antioxidants.

[32] Rajesh M., Mukhopadhyay P., Haskó G., Liaudet L., Mackie K., Pacher P. (2010). Cannabinoid-1 receptor activation induces reactive oxygen species-dependent and -independent mitogen-activated protein kinase activation and cell death in human coronary artery endothelial cells. Br. J. Pharmacol..

[33] Mukhopadhyay P., Horváth B., Rajesh M., Matsumoto S., Saito K., Bátkai S., Patel V., Tanchian G., Gao R.Y., Cravatt B.F. (2011). Fatty acid amide hydrolase is a key regulator of endocannabinoid-induced myocardial tissue injury. Free Radic. Biol. Med..

[34] Rajesh M., Bátkai S., Kechrid M., Mukhopadhyay P., Lee W.S., Horváth B., Holovac E., Cinar R., Liaudet L., Mackie K. (2012). Cannabinoid 1 receptor promotes cardiac dysfunction, oxidative stress, inflammation, and fibrosis in diabetic cardiomyopathy. Diabetes.

[35] Montecucco F., Lenglet S., Braunersreuther V., Burger F., Pelli G., Bertolotto M., Mach F., Steffens S. (2009). CB_2_ cannabinoid receptor activation is cardioprotective in a mouse model of ischemia/reperfusion. J. Mol. Cell. Cardiol..

[36] Mukhopadhyay P., Rajesh M., Pan H., Patel V., Mukhopadhyay B., Batkai S., Gao B., Haskó G., Pacher P. (2010). Cannabinoid-2 receptor limits inflammation, oxidative/nitrosative stress, and cell death in nephropathy. Free Radic. Biol. Med..

[37] Sun H.J., Lu Y., Wang H.W., Zhang H., Wang S.R., Xu W.Y., Fu H.L., Yao X.Y., Yang F., Yuan H.B. (2017). Activation of endocannabinoid receptor 2 as a mechanism of propofol pretreatment-induced cardioprotection against ischemia-reperfusion injury in rats. Oxid. Med. Cell. Longev..

[38] Duerr G.D., Heinemann J.C., Kley J., Eichhorn L., Frede S., Weisheit C., Wehner S., Bindila L., Lutz B., Zimmer A. (2019). Myocardial maladaptation to pressure overload in CB2 receptor-deficient mice. J. Mol. Cell. Cardiol..

[39] Matyas C., Erdelyi K., Trojnar E., Zhao S., Varga Z.V., Paloczi J., Mukhopadhyay P., Nemeth B.T., Haskó G., Cinar R. (2019). Interplay of liver-heart inflammatory axis and cannabinoid 2 receptor signalling in an experimental model of hepatic cardiomyopathy. Hepatology.

[40] Rajaraman G., Simcocks A., Hryciw D.H., Hutchinson D.S., McAinch A.J. (2016). G protein coupled receptor 18: A potential role for endocannabinoid signaling in metabolic dysfunction. Mol. Nutr. Food Res..

[41] Matouk A.I., Taye A., El-Moselhy M.A., Heeba G.H., Abdel-Rahman A.A. (2017). The effect of chronic activation of the novel endocannabinoid receptor GPR18 on myocardial function and blood pressure in conscious rats. J. Cardiovasc. Pharmacol..

[42] McPartland J.M., Duncan M., Di Marzo V., Pertwee R.G. (2015). Are cannabidiol and Δ^9^-tetrahydrocannabivarin negative modulators of the endocannabinoid system? A systematic review. Br. J. Pharmacol..

[43] Leishman E., Manchanda M., Thelen R., Miller S., Mackie K., Bradshaw H.B. (2018). Cannabidiol’s upregulation of N-acyl ethanolamines in the central nervous system requires N-acyl phosphatidyl ethanolamine-specific phospholipase D. Cannabis Cannabinoid Res..

[44] Bisogno T., Hanuš L., De Petrocellis L., Tchilibon S., Ponde D.E., Brandi I., Moriello A.S., Davis J.B., Mechoulam R., Di Marzo V. (2001). Molecular targets for cannabidiol and its synthetic analogues: Effect on vanilloid VR1 receptors and on the cellular uptake and enzymatic hydrolysis of anandamide. Br. J. Pharmacol..

[45] De Petrocellis L., Ligresti A., Moriello A.S., Allarà M., Bisogno T., Petrosino S., Stott C.G., Di Marzo V. (2011). Effects of cannabinoids and cannabinoid-enriched Cannabis extracts on TRP channels and endocannabinoid metabolic enzymes. Br. J. Pharmacol..

[46] Toczek M., Malinowska B. (2018). Enhanced endocannabinoid tone as a potential target of pharmacotherapy. Life Sci..

[47] Schloss M.J., Horckmans M., Guillamat-Prats R., Hering D., Lauer E., Lenglet S., Weber C., Thomas A., Steffens S. (2019). 2-Arachidonoylglycerol mobilizes myeloid cells and worsens heart function after acute myocardial infarction. Cardiovasc. Res..

[48] Biernacki M., Łuczaj W., Jarocka-Karpowicz I., Ambrożewicz E., Toczek M., Skrzydlewska E. (2018). The effect of long-term administration of fatty acid amide hydrolase inhibitor URB597 on oxidative metabolism in the heart of rats with primary and secondary hypertension. Molecules.

[49] Biernacki M., Malinowska B., Timoszuk M., Toczek M., Jastrząb A., Remiszewski P., Skrzydlewska E. (2018). Hypertension and chronic inhibition of endocannabinoid degradation modify the endocannabinoid system and redox balance in rat heart and plasma. Prostaglandins Other Lipid Mediat..

[50] Wheal A.J., Jadoon K., Randall M.D., O’Sullivan S.E. (2017). In vivo cannabidiol treatment improves endothelium-dependent vasorelaxation in mesenteric arteries of Zucker diabetic fatty rats. Front. Pharmacol..

[51] Lee W.S., Erdelyi K., Matyas C., Mukhopadhyay P., Varga Z.V., Liaudet L., Haskó G., Čiháková D., Mechoulam R., Pacher P. (2016). Cannabidiol limits T cell-mediated chronic autoimmune myocarditis: Implications to autoimmune disorders and organ transplantation. Mol. Med..

[52] Pastor A., Farré M., Fitó M., Fernandez-Aranda F., de la Torre R. (2014). Analysis of ECs and related compounds in plasma: Artifactual isomerization and ex vivo enzymatic generation of 2-MGs. J. Lipid Res..

[53] Alhouayek M., Bottemanne P., Makriyannis A., Muccioli G.G. (2017). N-acylethanolamine-hydrolyzing acid amidase and fatty acid amide hydrolase inhibition differentially affect N-acylethanolamine levels and macrophage activation. Biochim. Biophys. Acta Mol. Cell Biol. Lipids.

[54] Wortley M.A., Adcock J.J., Dubuis E.D., Maher S.A., Bonvini S.J., Delescluse I., Kinloch R., McMurray G., Perros-Huguet C., Papakosta M. (2017). Targeting fatty acid amide hydrolase as a therapeutic strategy for antitussive therapy. Eur. Respir. J..

[55] Shearer J.A., Coker S.J., Carswell H.V.O. (2018). Detrimental effects of 2-arachidonoylglycerol on whole blood platelet aggregation and on cerebral blood flow after a focal ischemic insult in rats. Am. J. Physiol. Heart Circ. Physiol..

[56] Jehle J., Schöne B., Bagheri S., Avraamidou E., Danisch M., Frank I., Pfeifer P., Bindila L., Lutz B., Lütjohann D. (2018). Elevated levels of 2-arachidonoylglycerol promote atherogenesis in ApoE^-/-^ mice. PLoS ONE.

[57] Chanda D., Oligschlaeger Y., Geraets I., Liu Y., Zhu X., Li J., Nabben M., Coumans W., Luiken J., Glatz J.F.C. (2017). 2-Arachidonoylglycerol ameliorates inflammatory stress-induced insulin resistance in cardiomyocytes. J. Biol. Chem..

[58] Su H.F., Samsamshariat A., Fu J., Shan Y.X., Chen Y.H., Piomelli D., Wang P.H. (2006). Oleylethanolamide activates Ras-Erk pathway and improves myocardial function in doxorubicin-induced heart failure. Endocrinology.

[59] Mattace Raso G., Simeoli R., Russo R., Santoro A., Pirozzi C., d’Emmanuele di Villa Bianca R., Mitidieri E., Paciello O., Pagano T.B., Orefice N.S. (2013). N-Palmitoylethanolamide protects the kidney from hypertensive injury in spontaneously hypertensive rats via inhibition of oxidative stress. Pharmacol. Res..

[60] Irvine R.J., White J., Chan R. (1997). The influence of restraint on blood pressure in the rat. J. Pharmacol. Toxicol. Methods.

[61] Piomelli D., Tarzia G., Duranti A., Tontini A., Mor M., Compton T.R., Dasse O., Monaghan E.P., Parrott J.A., Putman D. (2006). Pharmacological profile of the selective FAAH inhibitor KDS-4103 (URB597). CNS Drug Rev..

[62] Li D., Chen B.M., Peng J., Zhang Y.S., Li X.H., Yuan Q., Hu C.P., Deng H.W., Li Y.J. (2009). Role of anandamide transporter in regulating calcitonin gene-related peptide production and blood pressure in hypertension. J. Hypertens..

[63] Malinowska B., Toczek M., Pędzińska-Betiuk A., Schlicker E. (2019). Cannabinoids in arterial, pulmonary and portal hypertension—Mechanisms of action and potential therapeutic significance. Br. J. Pharmacol..

[64] Ho W.S., Barrett D.A., Randall M.D. (2008). ‘Entourage’ effects of N-palmitoylethanolamide and N-oleoylethanolamide on vasorelaxation to anandamide occur through TRPV1 receptors. Br. J. Pharmacol..

[65] Malinowska B., Baranowska-Kuczko M., Schlicker E. (2012). Triphasic blood pressure responses to cannabinoids: Do we understand the mechanism?. Br. J. Pharmacol..

[66] Iffland K., Grotenhermen F. (2017). An update on safety and side sffects of sannabidiol: A review of clinical data and relevant animal studies. Cannabis Cannabinoid Res..

[67] Pędzińska-Betiuk A., Weresa J., Toczek M., Baranowska-Kuczko M., Kasacka I., Harasim-Symbor E., Malinowska B. (2017). Chronic inhibition of fatty acid amide hydrolase by URB597 produces differential effects on cardiac performance in normotensive and hypertensive rats. Br. J. Pharmacol..

[68] Kamon T., Kaneko H., Itoh H., Kiriyama H., Mizuno Y., Morita H., Yamamichi N., Komuro I. (2019). Gender-specific association between the blood pressure category according to the updated ACC/AHA guidelines for hypertension and cardio-ankle vascular index: A community-based cohort study. J. Cardiol..

[69] Mozos I. (2014). Arrhythmia risk and obesity. J. Mol. Genet. Med..

[70] Gasparova I., Kubatka P., Opatrilova R., Caprnda M., Filipova S., Rodrigo L., Malan L., Mozos I., Rabajdova M., Nosal V. (2017). Perspectives and challenges of antioxidant therapy for atrial fibrillation. Naunyn Schmiedeberg Arch. Pharmacol..

[71] Mozos I. (2014). Laboratory markers of ventricular arrhythmia risk in renal failure. Biomed. Res. Int..

[72] Lam P.M., Marczylo T.H., El-Talatini M., Finney M., Nallendran V., Taylor A.H., Konje J.C. (2008). Ultra performance liquid chromatography tandem mass spectrometry method for the measurement of anandamide in human plasma. Anal. Biochem..

[73] Luque-Córdoba D., Calderón-Santiago M., Luque de Castro M.D., Priego-Capote F. (2018). Study of sample preparation for determination of endocannabinoids and analogous compounds in human serum by LC-MS/MS in MRM mode. Talanta.

[74] Siegmund S.V., Seki E., Osawa Y., Uchinami H., Cravatt B.F., Schwabe R.F. (2006). Fatty acid amide hydrolase determines anandamide-induced cell death in the liver. J. Biol. Chem..

[75] Ulloa N.M., Deutsch D.G. (2010). Assessment of a spectrophotometric assay for monoacylglycerol lipase activity. AAPS J..

[76] Lessard S.J., MacDonald T.L., Pathak P., Han M.S., Coffey V.G., Edge J., Rivas D.A., Hirshman M.F., Davis R.J., Goodyear L.J. (2018). JNK regulates muscle remodeling via myostatin/SMAD inhibition. Nat. Commun..

[77] Aebi H. (1984). Catalase in vitro. Methods Enzymol..

[78] Mize C.E., Langdon R.G. (1962). Hepatic glutathione reductase. I. Purification and general kinetic properties. J. Biol. Chem..

[79] Paglia D.E., Valentine W.N. (1967). Studies on the quantitative and qualitative characterization of erythrocyte glutathione peroxidase. J. Lab. Clin. Med..

[80] Sykes J.A., McCormack F.X., O’Brien T.J. (1978). A preliminary study of the superoxide dismutase content of some human tumors. Cancer Res..

[81] Vatassery G.T., Brin M.F., Fahn S., Kayden H.J., Traber M.G. (1988). Effect of high doses of dietary vitamin E on the concentrations of vitamin E in several brain regions, plasma, liver, and adipose tissue of rats. J. Neurochem..

[82] Maeso N., García-Martínez D., Rupérez F.J., Cifuentes A., Barbas C. (2005). Capillary electrophoresis of glutathione to monitor oxidative stress and response to antioxidant treatments in an animal model. J. Chromatogr. B.

[83] Levine R.L., Garland D., Oliver C.N., Amici A., Climent I., Lenz A.G., Ahn B.W., Shaltiel S., Stadtman E.R. (1990). Determination of carbonyl content in oxidatively modified proteins. Methods Enzymol..

[84] Luo X.P., Yazdanpanah M., Bhooi N., Lehotay D.C. (1995). Determination of aldehydes and other lipid peroxidation products in biological samples by gas chromatography-mass spectrometry. Anal. Biochem..

[85] Christie W.W., Christie W.W. (1993). Preparation of ester derivatives of fatty acids for chromatographic analysis. Advances in Lipid Methodology—Two.

